# The effects of soil phosphorus and zinc availability on plant responses to mycorrhizal fungi: a physiological and molecular assessment

**DOI:** 10.1038/s41598-019-51369-5

**Published:** 2019-10-16

**Authors:** Thi Diem Nguyen, Timothy R. Cavagnaro, Stephanie J. Watts-Williams

**Affiliations:** 1 The School of Agriculture, Food & Wine and The Waite Research Institute, The University of Adelaide, Glen Osmond, South Australia 5064 Australia; 2 The Australian Research Council Centre of Excellence in Plant Energy Biology, The University of Adelaide, Glen Osmond, South Australia 5064 Australia; 3 Institute of Biotechnology, Hue University, Provincial Road 10, Ngoc Anh, Phu Thuong, Phu Vang, Thua Thien Hue 49000 Vietnam

**Keywords:** Plant molecular biology, Arbuscular mycorrhiza

## Abstract

The positive effects of arbuscular mycorrhizal fungi (AMF) have been demonstrated for plant biomass, and zinc (Zn) and phosphorus (P) uptake, under soil nutrient deficiency. Additionally, a number of Zn and P transporter genes are affected by mycorrhizal colonisation or implicated in the mycorrhizal pathway of uptake. However, a comprehensive study of plant physiology and gene expression simultaneously, remains to be undertaken. *Medicago truncatula* was grown at different soil P and Zn availabilities, with or without inoculation of *Rhizophagus irregularis*. Measures of biomass, shoot elemental concentrations, mycorrhizal colonisation, and expression of Zn transporter (*ZIP*) and phosphate transporter (*PT*) genes in the roots, were taken. Mycorrhizal plants had a greater tolerance of both P and Zn soil deficiency; there was also evidence of AMF protecting plants against excessive Zn accumulation at high soil Zn. The expression of all *PT* genes was interactive with both P availability and mycorrhizal colonisation. *MtZIP5* expression was induced both by AMF and soil Zn deficiency, while *MtZIP2* was down-regulated in mycorrhizal plants, and up-regulated with increasing soil Zn concentration. These findings provide the first comprehensive physiological and molecular picture of plant-mycorrhizal fungal symbiosis with regard to soil P and Zn availability. Mycorrhizal fungi conferred tolerance to soil Zn and P deficiency and this could be linked to the induction of the *ZIP* transporter gene *MtZIP5*, and the PT gene *MtPT4*.

## Introduction

Zinc (Zn) is important both for agricultural production and for human development. A deficiency of Zn can seriously affect plant and human development because Zn is a regulatory co-factor and structural constituent in proteins and enzymes involved in many biochemical pathways^1,2^. Soil Zn deficiency affects millions of hectares of cropland worldwide, and is particularly prevalent in developing countries, and in the cereal-growing regions of Australia^2,3^. In addition, approximately one third of the global population suffers from an inadequate dietary intake of Zn^4–6^. Therefore, increasing the level of Zn uptake by crops - known as biofortification - is a subject of considerable international interest^7–9^.

Phosphorus (P) is one of the most important macronutrients for plant growth. Soil P deficiency leads to reduced plant nutrient uptake, prolonged maturity stage^10,11^, and affects enzyme activity and many signal transduction cascades^12^. However, the primary source of P fertilizer (phosphate rock) for plant growth is a finite resource, and thus is becoming depleted over time because of demand from the agricultural sector^13,14^. To effectively manage the available levels of soil Zn and P, an advanced understanding of plant root uptake capacity of nutrients is required, in particular, of plant membrane transporters related to P and Zn transport^15^. Additionally, it is important to understand factors that affect P and Zn transport gene regulation, such as associations with arbuscular mycorrhizal fungi (AMF), which can enhance plant nutrient uptake^16^.

AMF form associations with more than 80% of flowering plant species, and form part of the function of plant root systems^17,18^. AMF colonise the root cortex, and can extend their hyphal network into the surrounding soil environment^19,20^. These external hyphae contribute to plant uptake of P and Zn, as well as other mineral nutrient including iron (Fe), calcium (Ca), and copper (Cu)^18^. Previous research has demonstrated that AMF can enhance plant Zn uptake, sometimes leading to a boost in plant growth and Zn concentration in plant tissue^21,22^. Furthermore, P concentration in the shoots and roots of mycorrhizal plants can be significantly higher than in non-mycorrhizal plants grown under soil P deficiency^23–26^. Additionally, AMF can reduce plant heavy metal uptake (Zn, copper (Cu), lead (Pb), arsenic (As)) in contaminated soils, thereby protecting plants from toxic effects^27,28^. Studies on red clover and tomato, respectively, have established that Zn content uptake in shoots and roots decreased substantially under high soil Zn concentration when plants were colonised by AMF^29,30^.

There are two pathways for plant uptake of nutrients from soil: via root epidermal cells (direct pathway; DPU), and via associations with arbuscular mycorrhizal fungi (mycorrhizal pathway; MPU). In plants, Zn is taken up from the rhizosphere as Zn^2+^ via ZIP (Zrt, Irt-like Protein) membrane transporters^31^. Some ZIP transporters have the potential to also transport Fe^2+^ and Mn^2+^ ^31,32^. Several studies have focused on characterising the ZIP transporter family in different plant species, including barley^32–35^, rice^36^, potato, and *Arabidopsis thaliana*^31^. In *Medicago truncatula* (Medicago), four ZIP transporters - MtZIP1, MtZIP2, MtZIP5 and MtZIP6 - are able to transport Zn^2+^, as confirmed by yeast complementation assays, have been identified^37,38^. As such, Medicago provide a model plant species for studies of mycorrhizal impacts on plant Zn (and P, see below) nutrition. Additionally, MtZIP2 has been localised to the plasma membrane in onion epidermal cells^39^. Further work is required to discover whether any of these ZIP transporters are implicated in the mycorrhizal transport of Zn into plants (via the MPU).

In terms of plant P uptake, the phosphate transporters (PTs) MtPT1, MtPT2, and MtPT3 are involved in the DPU for P uptake, and are closely related to low-affinity P transporters, belonging to the Pht1 family^40,41^. The genes encoding these PTs are generally highly expressed when plants are growing under low soil P conditions. However, in roots colonised by AMF, *MtPT1* and *MtPT2* are down-regulated significantly as the symbiosis develops^26,42,43^. Furthermore, one of the PTs in *M*. *truncatula* (MtPT4) has been demonstrated to be a mycorrhiza-induced phosphate transporter^44^. *MtPT4* is expressed exclusively in mycorrhizal roots, and specifically, in cells containing arbuscules^44^. Loss of MtPT4 function in plants leads to impairment of the mycorrhizal symbiosis, because it causes mature arbuscules to degenerate, resulting in premature arbuscular death^45,46^. In addition, MtPT8 from the Pht1 family has also been identified to be induced upon formation of the mycorrhizal symbiosis, and contribute to the uptake of P ions released by the fungal membranes^47–49^.

A phosphate starvation-induced (PSI) gene in *M*. *truncatula*, *MT4*^26,50^, has been shown to be involved in P accumulation in plants, and is down-regulated in response to both P fertilisation and to mycorrhizal colonization^51^. More recently, another gene (*LysoPhosphatidylCholine AcylTransferase* 1; *AtLPCAT1*) has been demonstrated to be involved in the accumulation of P in the shoot under soil Zn deficiency in *A*. *thaliana* (a non-mycorrhizal species)^52^. Therefore, these genes are also of interest with regards to plant responses to P and Zn deficiency in the present study.

In *M*. *truncatula*, there is evidence that mycorrhizal inoculation affects plant P and Zn uptake at different soil P concentrations, and at a range of soil Zn concentrations, ranging from deficient to toxic^30,53,54^. However, there presently exists a lack of research focusing on the three-way interaction between soil P and Zn availabilities and AMF inoculation, that also addresses potential underlying molecular mechanisms. Therefore, the current study aimed to link the mycorrhizal effects under variable P and Zn soil conditions, on whole plant physiology, with expression of molecular markers for Zn and P uptake. Specifically, there were several hypotheses related to the experiment:

1. That the effects of soil Zn and P availability were interactive with AMF function, and
2. That the effects of AMF on plant nutrition and gene expression could be linked:
  - In relation to the dual role of AMF at low and high soil Zn availability, and
  - In relation to the role of AMF in improving P uptake at low soil P availability

## Material and Methods

Field soil was collected from the Mallala region of South Australia, and had a pH of 7.1 and plant-available (Colwell) P concentration of 22 mg kg^−1^. Soil was sieved to <2 mm, and both fine sand and soil were autoclaved twice, then dried, before being mixed in a ratio of 9:1 sand/soil^53^. The sand/soil mix was further mixed with 10% (140 g per pot) of either a *Rhizophagus irregularis* WFVAM10 or a non-mycorrhizal mock inoculum (see below) to a total mass of 1.4 kg per pot. The *R*. *irregularis* inoculum was added as a mixture of dry soil, spores, external AMF hyphae and root fragments of *Trifolium subterraneum* L. (clover) cv. Mt. Barker pot cultures. The control, a mock inoculum, was a mixture of dry soil and root fragments of *Trifolium subterraneum* L. (clover) cv. Mt. Barker pots that had not been inoculated with AMF.

To establish soil Zn addition treatments, ZnSO_4_.7H_2_O solution was added to the soil/sand mix at the rates of 0, 5, 10, and 20 mg Zn kg^−1^ soil. This resulted in four soil Zn treatments, with DTPA-extractable Zn concentrations of 0.3, 4.0, 5.8, and 15.0 mg Zn kg^−1^, referred to hereafter as Zn0, Zn5, Zn10, and Zn20, respectively. Phosphorus treatments were established by adding K_2_HPO_4_ solution to the soil at concentrations of 0, 20, and 50 mg P kg^−1^ soil, resulting in three plant-available (Colwell) P concentrations: 4.4, 13.8, and 31.8 mg P kg^−1^, referred to hereafter as P0, P20, and P50, respectively. Plastic, non-draining pots were then filled with the prepared soils. Each soil Zn addition treatment, soil P addition treatment, and AMF treatment were combined in a factorial manner, so that in total there were 24 treatments, each with five biological replicates, giving a total of 120 pots.

*Medicago truncatula* cv. Jemalong A17 seeds were scarified using fine sandpaper, then surface-sterilised for 5 minutes by shaking in 10% sodium hypochlorite solution (NaClO). Then, seeds were washed and rinsed with reserve osmosis (RO) water before being placed onto moist filter paper in a Petri dish. The Petri dishes then were sealed with Parafilm, covered with aluminium foil and kept at 4 °C for four days. Following that, dishes were transferred to the bench and left covered at room temperature for one day, before being uncovered, unsealed, and left for a further three days while being provided water daily. When green cotyledons had emerged, seedlings were transplanted to the prepared soils (one plant per pot) following Watts-Williams, *et al*.^53^.

The *M*. *truncatula* plants were grown in a controlled environment glasshouse at the University of Adelaide, Waite campus, between March-April 2018 (Austral Autumn). Plants were watered three to four times per week (based on plant demand) with RO water to 10% of the soil weight. They also were nutritionally supplemented once per week with 10 mL of a modified Long-Ashton solution that omitted Zn and P^53^. Furthermore, to avoid *Rhizobia* bacteria forming nodules, plants also were supplemented with nitrogen as NH_4_NO_3,_ solution to a total of 80 mg N per plant over the growing period. Plant position on the glasshouse bench was randomised, and re-randomised once per week.

All plants were destructively harvested 36 days post-transplantation. Shoots were cut at the soil level, then were weighed for fresh shoot biomass. Roots were washed thoroughly of soil and were then weighed for fresh root biomass, before subsamples were taken for mycorrhizal colonisation and gene expression analysis. The root subsamples were immediately flash frozen in liquid nitrogen and then stored at −80 °C (for gene expression studies; see below), or placed into 50% ethanol (EtOH) solution (for quantification of mycorrhizal colonisation; see below). Nodulation was observed in only three plants, indicating that N fertilisation was generally effective at suppressing nodulation by rhizobia. Both fresh shoot and remaining fresh root biomass were then dried at 60 °C for at least 48 hours before dry biomass was determined. Next, dry shoot biomass was ground to homogenise, then a weighed sub-sample was used for digestion using a 4:1 (*v*/*v*) mix of nitric acid (HNO_3_) and hydrogen peroxide (H_2_O_2_). Acid digests were then diluted with RO water, before being analysed for nutrient concentrations (including Zn, P and other nutrients) by Inductively Coupled Plasma-Atomic Emission Spectroscopy analytical technique (ICP-AES; Avio 200 ICP Optical Emission Spectrometer).

Fresh root subsamples stored in 50% EtOH were rinsed in RO water and then cleared by submerging in 10% (w/v) potassium hydroxide (KOH) at room temperature for seven days. The cleared roots were then rinsed again with RO water before being stained in a solution of 5% of ink in vinegar^53,55^ at 60 °C for 10 minutes. The root samples were then rinsed and de-stained in RO water for 24 hours, before being transferred to a 50% glycerol solution for storage and microscope assessment. Mycorrhizal colonisation was determined on the stained root samples under a dissection microscope using the gridline intersect method^56^.

For gene expression analysis, transcript levels of chosen *M*. *truncatula ZIP*s, *PT*s, and other genes were determined by quantitative real-time PCR (qPCR). The *MtLPCAT1* gene was determined as the nearest *M*. *truncatula* orthologue of the *AtLPCAT1* gene sequences by BLAST (https://phytozome.jgi.doe.gov/pz/portal.html#!search?show=BLAST; last accessed 15 October, 2018). The flash-frozen root subsamples were ground to a fine powder under liquid nitrogen. RNA was then extracted using a Spectrum Plant Total RNA kit (Sigma) including an on-column DNase treatment step (Sigma). Following this, the RNA yield and quality was quantified by Nanodrop, and the iScript cDNA synthesis kit (Bio-Rad) was used to synthesise cDNA from 800 ng of RNA. The expression of *MtZIP* genes, *MtPT* genes, and other genes-of-interest, as well as fungal α-tubulin gene (a marker gene for *R*. *irregularis* biomass in roots), were quantified by qPCR (QuantStudio 12 K Flex Real-Time PCR system, Applied Biosystems), using forward and reverse primers designed to target the specific genes (Supplementary Information Table 1). The primer pair for *MtASPP* amplification spanned an exon-exon boundary in the gene and were thus used to confirm there was no genomic DNA contamination following RNA extraction. Expression of each gene-of-interest was then normalised to the geometric mean of the expression of three *M*. *truncatula* housekeeping genes.

Data were checked for the assumption of normal distribution using Genstat (19^th^ edition), and any non-normal data (*p* < 0.05) were log- or square root-transformed to conform to the assumption of normality. In Figures, presented values are non-normalised data. For the physiological and gene expression data, the response variables were subjected to three-factor analysis of variance (ANOVA), with *Mycorrhiza*, *Zn*, and *P* as treatment factors. Following ANOVA, the mycorrhizal and control treatment means were compared at each soil Zn and P treatment, respectively, by a Student’s *t*-test. For response variables where there was only mycorrhizal data (% mycorrhizal colonisation, *MtPT4*, *MtPT8*, and *R*. *irregularis* fungal α-tubulin expression), two-way ANOVA was applied, with *P* and *Zn* treatments the factors. Following ANOVA, where there was a significant main effect or interaction, comparisons between treatment means were made using Tukey’s honestly significant difference (HSD) *post hoc* test. The relationships between *R*. *irregularis* fungal α-tubulin and *MtPT4* expression were analysed by regression in the mycorrhizal samples only. All statistical analyses were performed using Genstat (19^th^ Edition) or Microsoft Excel 2016 (Office 365 ProPlus).

## Results

### Mycorrhiza, zinc and phosphorus interact to affect plant biomass and nutrition (Hypothesis 1)

**Figure 1:**
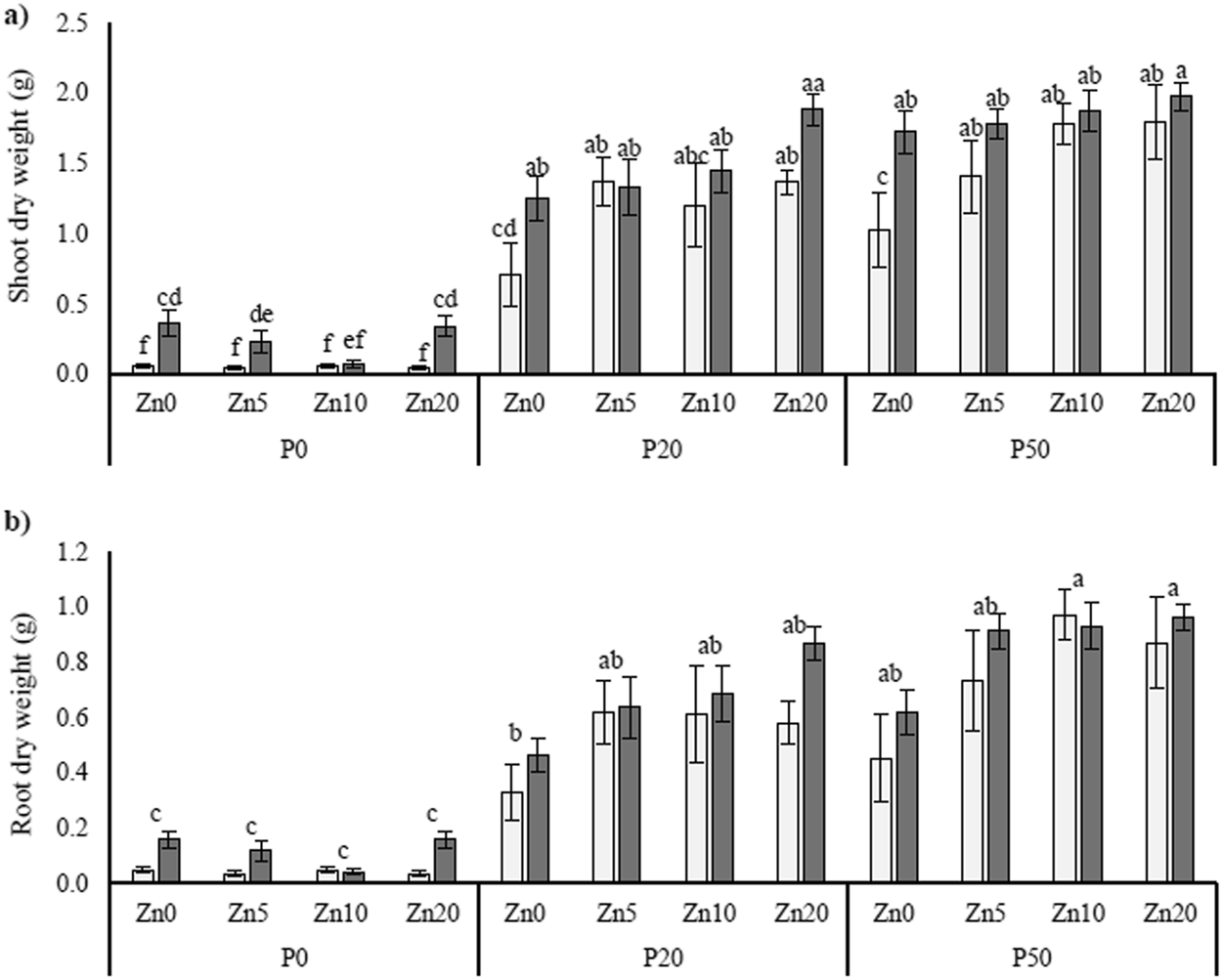
Shoot dry weights (**a**) and root dry weights (**b**) of *Medicago truncatula* plants inoculated with the AMF *R*. *irregularis* (grey bars) or mock-inoculated (white bars) and grown at different soil Zn (Zn0-Zn20) and P (P0-P50) concentrations. Values are mean ± SEM, *n* = 5. Means labelled with the same letter were not significantly different at the P < 0.05 level (Tukey’s HSD), see Table 1 for details of ANOVA results.

In general, shoot dry weight (SDW) and root dry weight (RDW) increased dramatically with increasing soil P addition, with mean SDW values ranging from 0.042 g (mock-inoculated P0, Zn5) to 1.977 g (AMF-inoculated P50, Zn20) (Fig. 1a). In terms of AMF-inoculation, the SDW of the mycorrhizal plants were greater than the mock-inoculated plants when no P was applied, except in the Zn10 treatment; a similar result was found for the RDW (see Table 1 for ANOVA outcomes; Fig. 1b). Regardless of P application, the inoculated plants were also larger in terms of both shoots and roots, when no Zn was applied to the soil.

**Table 1:** Statistical outcomes of three-way ANOVA for a range of plant physiological variables, and expression of genes interest in the roots.

	AMF	P	Zn	AMF*P	AMF*Zn	P*Zn	AMF*P*Zn
Shoot dry weight	<0.001	<0.001	ns	<0.001	0.011	0.009	0.024
Root dry weight	<0.001	<0.001	ns	0.005	0.042	0.003	ns
Shoot P concentration	0.008	<0.001	ns	0.002	<0.001	ns	0.007
Shoot P content	<0.001	<0.001	ns	<0.001	<0.001	0.011	0.042
Shoot Zn concentration	<0.001	<0.001	<0.001	<0.001	0.005	0.011	0.025
Shoot Zn content	ns	<0.001	<0.001	ns	ns	<0.001	ns
*MtZIP2* expression	ns	0.005	<0.001	ns	ns	ns	ns
*MtZIP5* expression	<0.001	<0.001	<0.001	ns	<0.001	<0.001	0.013
*MtZIP6* expression	<0.001	0.029	<0.001	0.005	0.047	ns	0.019
*MtPT1* expression	<0.001	<0.001	<0.001	<0.001	0.005	0.002	ns
*MtLPCAT1* expression	<0.001	ns	0.008	ns	ns	ns	ns
*MtMT4* expression	<0.001	<0.001	0.025	<0.001	0.009	0.006	0.001

**Figure 2:**
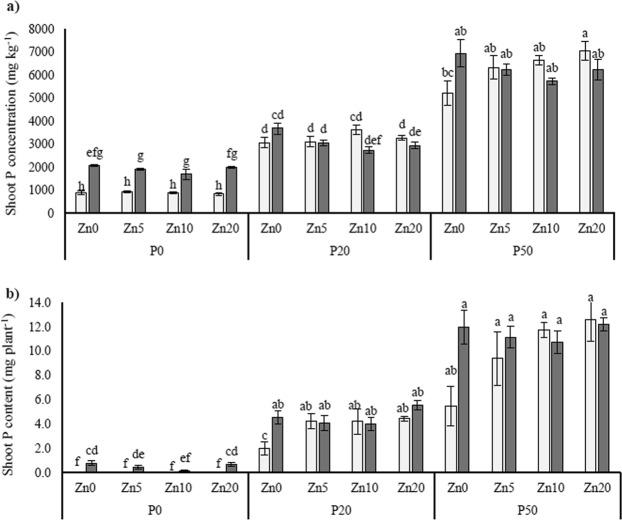
Shoot P concentration (**a**) and shoot P contents (**b**) in *Medicago truncatula* plants inoculated with the AMF *R*. *irregularis* (grey bars) or mock-inoculated (white bars), and grown at different soil Zn (Zn0-Zn20) and P (P0-P50) concentrations. Values are mean ± SEM, n = 5. Means labelled with the same letter were not significantly different at the P < 0.05 level (Tukey’s HSD), see Table 1 for details of ANOVA results.

**Figure 3:**
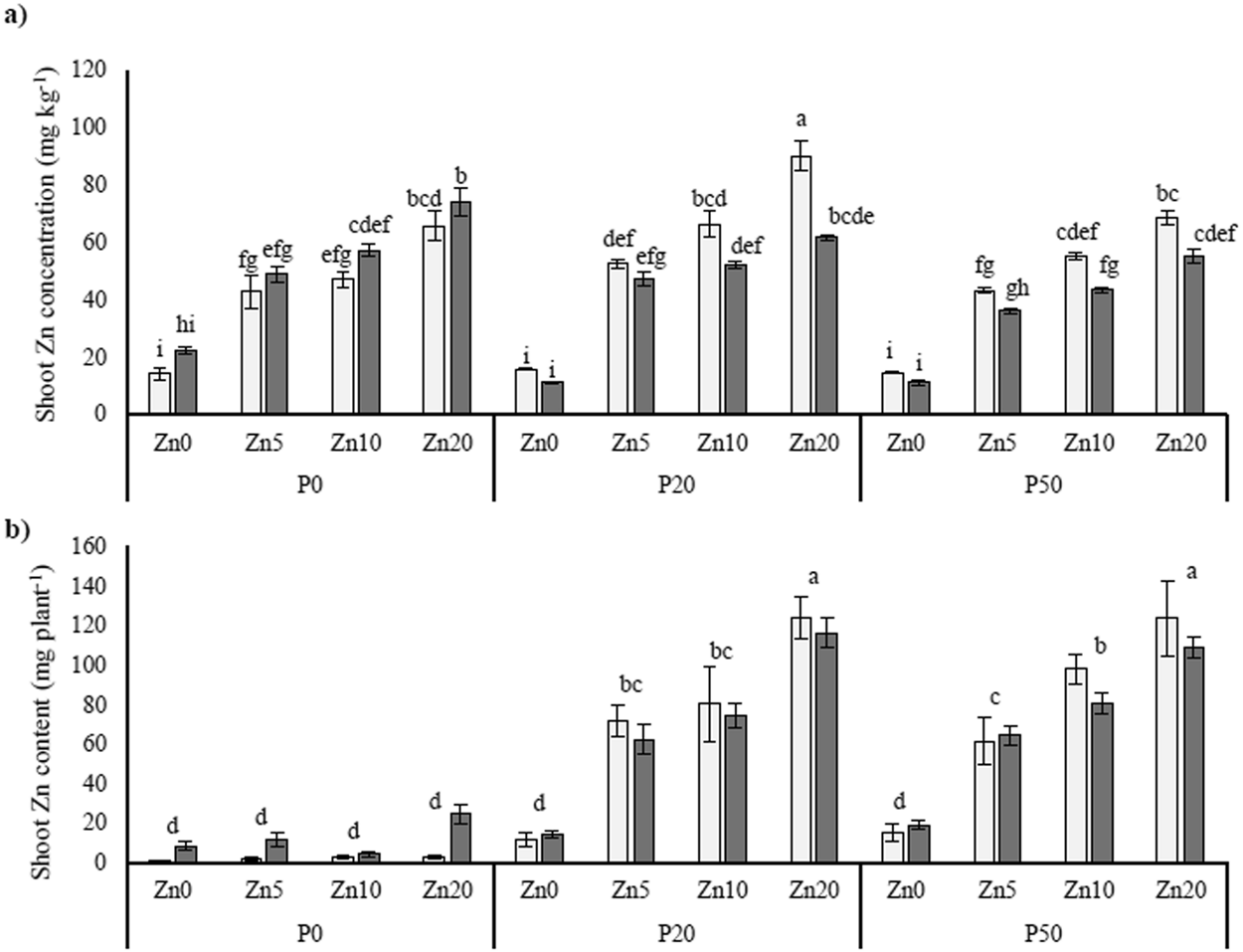
Shoot Zn concentration (**a**) and shoot Zn contents (**b**) in *Medicago truncatula* plants inoculated with the AMF *R*. *irregularis* (grey bars) or mock-inoculated (white bars), and grown at different soil Zn (Zn0-Zn20) and P (P0-P50) concentrations. Values are mean ± SEM, n = 5. Means labelled with the same letter were not significantly different at the P < 0.05 level (Tukey’s HSD), see Table 1 for details of ANOVA results.

Shoot P concentration (Fig. 2a) and shoot P contents (Fig. 2b) revealed similar statistical trends, and increased substantially with increasing soil P addition. Furthermore, shoot P concentration was significantly higher in the mycorrhizal plants than in the mock-inoculated plants when soil P was limiting (P0). Shoot Zn concentration (Fig. 3a) was similar between the mycorrhizal and mock-inoculated plants in all treatments, except for in the Zn20 P20 treatment where it was higher in the mock-inoculated plants. Shoot Zn contents (Fig. 3b), revealed an effect of increasing soil Zn, but was not affected by mycorrhizal inoculation.

### Mycorrhizal colonisation markers decrease under high phosphorus application

**Figure 4:**
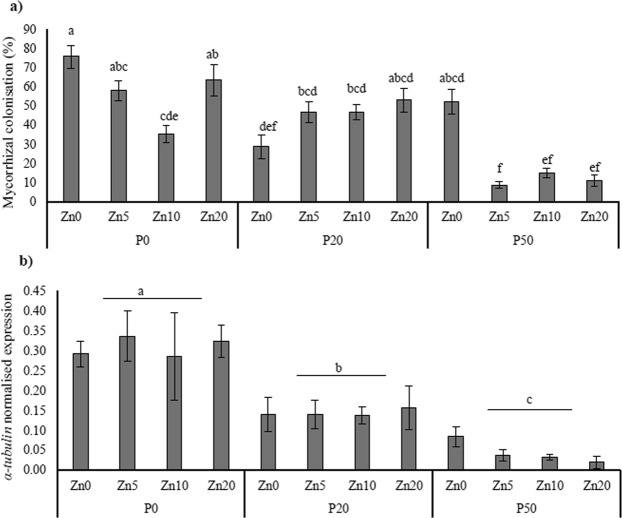
Root colonisation by arbuscular mycorrhizal fungal structures (**a**), and normalised expression of the AMF biomass marker gene α-tubulin (**b**) in the roots of *Medicago truncatula* plants inoculated with the AMF *R*. *irregularis*, and grown at different soil Zn (Zn0-Zn20) and P (P0-P50) concentrations. Values are mean ± SEM, n = 5. Means labelled with the same letter were not significantly different at the P < 0.05 level (Tukey’s HSD), see Table 2 for details of ANOVA results.

Mycorrhizal colonisation in AMF-inoculated roots was highest when Zn and P were the most limiting to the plant (P0, Zn0) (mean 75.8%; Fig. 4a), but decreased substantially with increasing soil P concentration to 8.7% in the P50, Zn5 treatment (see Table 2 for ANOVA outcomes). Mycorrhizal colonisation was also affected negatively by increasing soil Zn application, but only in the P0 and P50 treatments. The *R*. *irregularis* α-tubulin gene, a marker for live mycorrhizal fungal biomass in roots at the time of harvest, was expressed in a similar pattern to the root colonisation data (Fig. 4b) whereby expression decreased with increasing soil P application. There was no mycorrhizal colonisation observed in the roots of the mock-inoculated plants when examined by microscopy, nor expression of the *R*. *irregularis* α-tubulin gene in any of the mock-inoculated plants.

**Table 2:** Statistical outcomes of two-way ANOVA for mycorrhiza-specific variables.

	P	Zn	P*Zn
% mycorrhizal colonisation	<0.001	<0.001	<0.001
*R*. *irregularis α-tubulin* expression	<0.001	ns	ns
*MtPT8* expression	<0.001	ns	ns
*MtPT4* expression	<0.001	ns	ns

### *ZIP* gene expression was modulated by mycorrhiza and Zn-deficiency (Hypothesis 2a)

**Figure 5:**
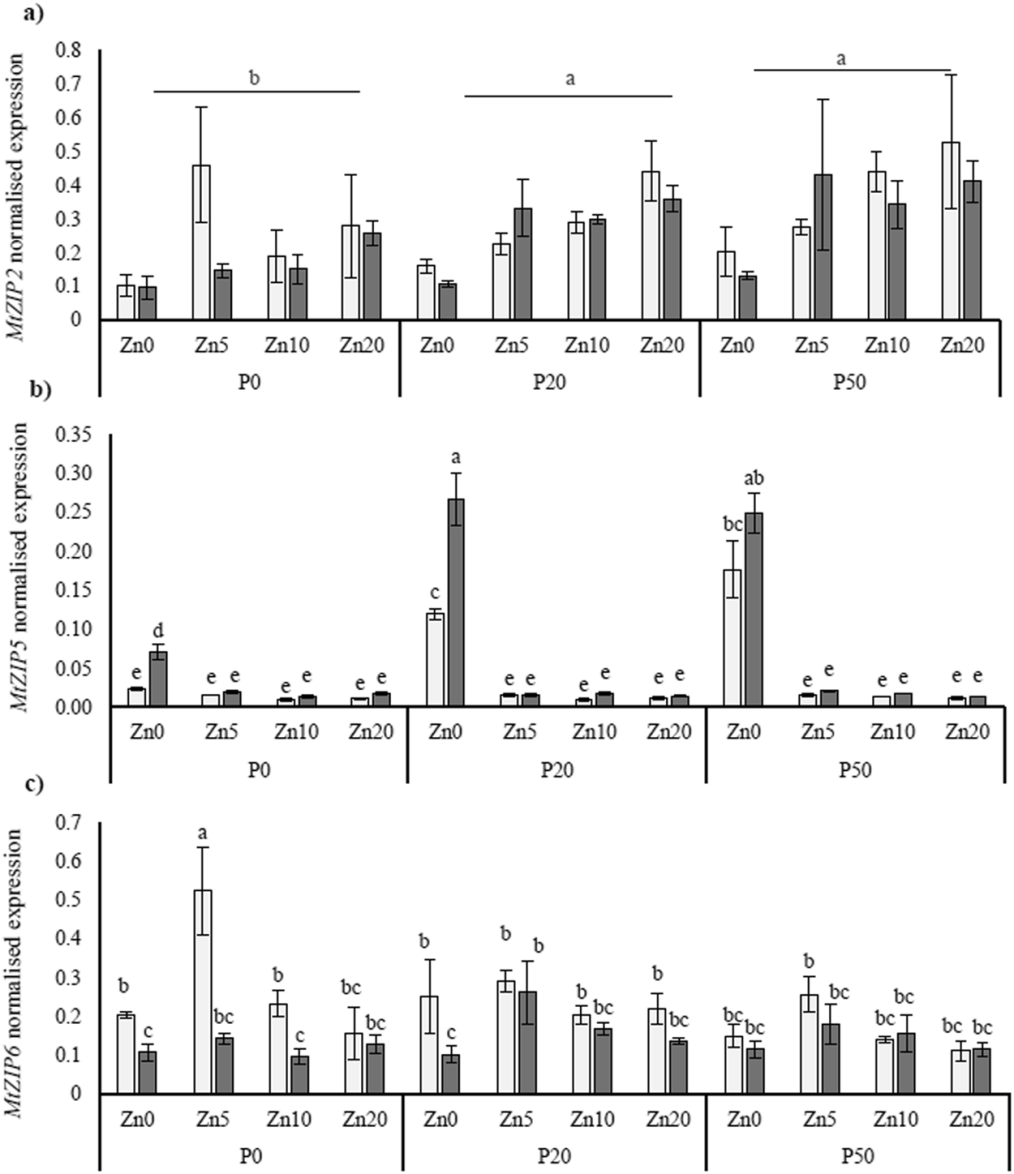
Normalised expression of the Zn-transporting *ZIP* transporter genes: *MtZIP2* (**a**), *MtZIP5* (**b**), and *MtZIP6* (**c**) in the roots of *Medicago truncatula* plants inoculated with the AMF *R*. *irregularis* (grey bars) or mock-inoculated (white bars), and grown at different soil Zn (Zn0-Zn20) and P (P0-P50) concentrations. Values are mean ± SEM, n = 5. Means labelled with the same letter were not significantly different at the P < 0.05 level (Tukey’s HSD), see Table 1 for details of ANOVA results.

The different *ZIP* genes were all affected by the Zn, P and AMF treatments in different ways, as follows. While the expression of *MtZIP2* was primarily affected by the P and Zn application treatments (Fig. 5a), the expression of *MtZIP5* and *MtZIP6* expression were affected by the interaction between P, Zn, and mycorrhizal colonisation. Specifically, the expression of *MtZIP2* increased between the lowest and the highest soil P treatments and, also increased between the lowest and highest soil Zn treatments. Expression of *MtZIP5* was induced at the lowest Zn treatment (Zn0), and was also higher in the mycorrhizal plants than in mock-inoculated plants at Zn0 (Fig. 5b). Conversely, expression of *MtZIP6* was significantly higher in the mock-inoculated plants than in the mycorrhizal plants in some treatments, but particularly where P and Zn additions were very low (Fig. 5c). The expression of *MtZIP1* was analysed, but was very low and highly variable between biological replicates, so is not presented here.

### Direct- and mycorrhizal-pathway *PT* genes display contrasting expression patterns (Hypothesis 2b)

**Figure 6:**
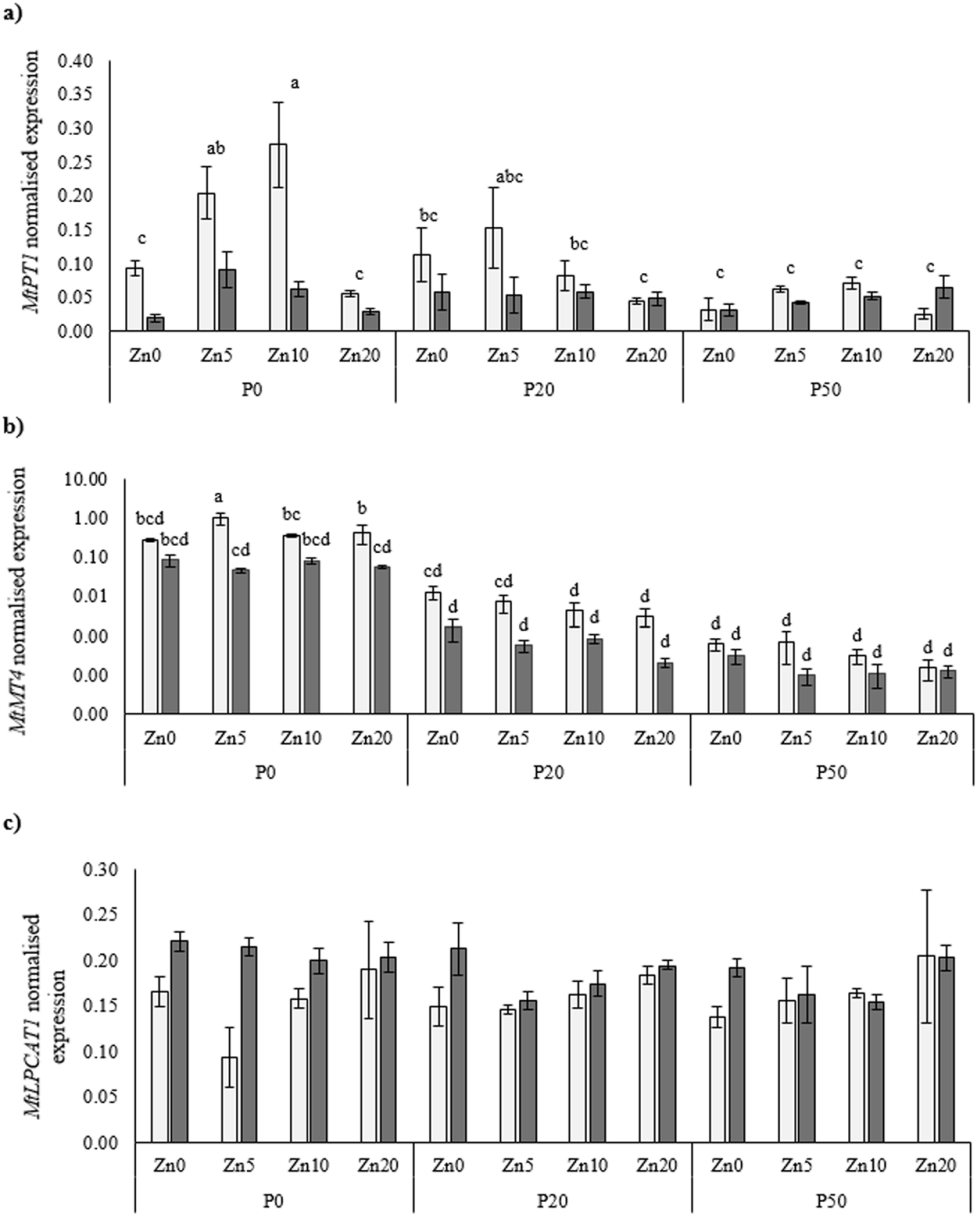
Normalised expression of the phosphate transporter gene *MtPT1* (**a**), a phosphate starvation-induced (PSI) gene *MtMT4* (presented on log-scale y-axis; **b**), and *MtLPCAT1* (**c**) in the roots of *Medicago truncatula* plants inoculated with the AMF *R*. *irregularis* (grey bars) or mock-inoculated (white bars), and grown at different soil Zn (Zn0-Zn20) and P (P0-P50) concentrations. Values are mean ± SEM, n = 5. Means labelled with the same letter were not significantly different at the P < 0.05 level (Tukey’s HSD), see Table 1 for details of ANOVA results.

The direct pathway *PT* gene *MtPT1* was down-regulated in the mycorrhizal plants (Fig. 6a), as was the P-starvation-induced gene *MtMT4* (Fig. 6b). The expression of both these genes was also repressed as soil P addition increased. Expression of *MtLPCAT1* was affected by mycorrhizal inoculation, and was lower in the mock-inoculated plants than the mycorrhizal plants (pooling P and Zn treatments; Fig. 6c).

**Figure 7:**
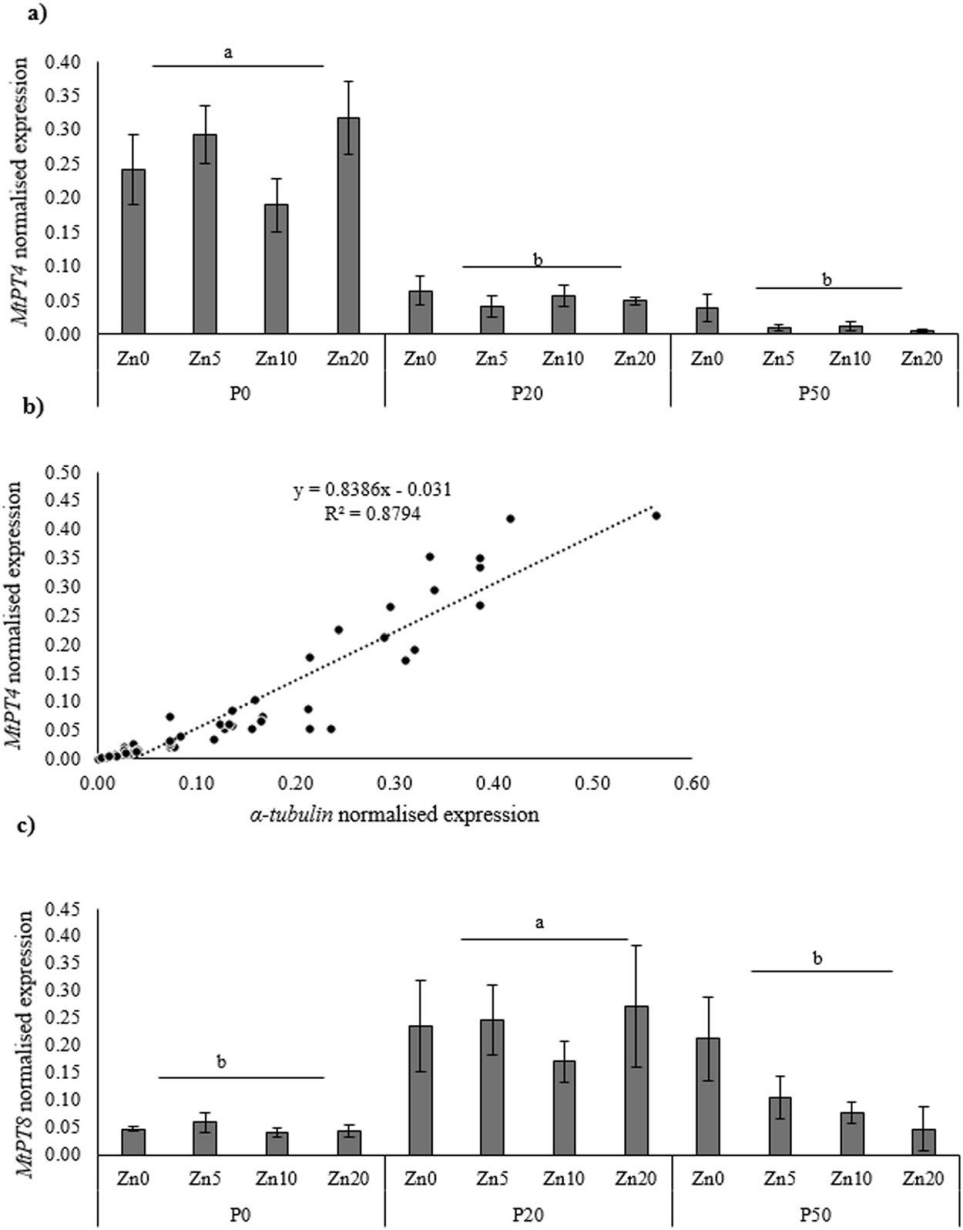
Normalised expression of the mycorrhiza-induced phosphate transporter (*PT*) gene *MtPT4* (**a**) correlation between the expression of the *R*. *irregularis* biomass marker gene *α-tubulin* and *MtPT4 (***b**), and expression of the mycorrhiza-induced PT *MtPT8* (**c**) in the roots of *Medicago truncatula* inoculated with the AMF *R*. *irregularis*, and grown at different soil Zn (Zn0-Zn20) and P (P0-P50) concentrations. Values are mean ± SEM, n = 5. Means labelled with the same letter were not significantly different at the P < 0.05 level (Tukey’s HSD), see Table 2 for details of ANOVA results.

Finally, *MtPT4* was expressed exclusively in the mycorrhizal roots, and there was a very clear reduction of expression as soil P addition increased (Fig. 7a), which correlated strongly and positively with *R*. *irregularis* α-tubulin expression (R^2^ = 0.8794) (Fig. 7b). In contrast, the expression of another AMF-induced PT, *MtPT8*, revealed a very different pattern to *MtPT4*, with increased expression between P0 and P20, and then decreased between P20 and P50 (Fig. 7c).

## Discussion

### Mycorrhizal plants are more tolerant of soil P and Zn deficiencies

The positive effects of forming mycorrhizal associations on plant growth are considered one of the main benefits of the symbiosis, and these effects have been studied extensively in various plant species^57–62^. In this study, the benefits of mycorrhizal fungal inoculation in terms of increased Medicago biomass were the greatest under low soil P concentration, which confirms earlier studies in this and other plant species^13,30,63^. In the two soil P addition treatments (P20, P50), there were smaller, albeit still positive, plant biomass responses to mycorrhizal inoculation. These smaller responses may be due to the sufficient levels of P in soil, which allowed plants to increase their biomass and nutrient uptake without the aid of mycorrhizal fungi. In an earlier study, it was demonstrated using radioisotope tracing that even where the biomass of mycorrhizal flax (*Linum usitatissium*) plants were not larger than mock-inoculated plants, the MPU was still active and transporting substantial amounts of P to the plant^64^. Thus, the growth response of a plant to mycorrhizal fungi, whether it is positive, neutral or negative, cannot be used to estimate the activity of the mycorrhizal pathway of P uptake. In the present study, the reduction in growth response was in line with the decreased percentage of mycorrhizal colonisation in the roots, when P was added to the soil, as demonstrated in previous studies in both the same plant and fungi species^26^ and in other species^14,65–68^. Consequently, the reduction in mycorrhizal colonisation and MGR with soil P addition points to a reduced contribution of P by the MPU; however, the actual activity of the MPU would need to be confirmed by radioisotope labelling.

With regards to soil Zn, colonisation by mycorrhizal fungi resulted in increased Medicago biomass under both soil Zn deficiency (Zn0) and soil Zn toxicity (Zn20); this result highlights the dual role of mycorrhizal fungi in ameliorating Zn stress for the host plant^53,69,70^. While the mycorrhizal plants thrived in soil with high levels of Zn addition (Zn20), the non-mycorrhizal plants experienced reduced growth and visual symptoms of Zn toxicity in the leaves, as in earlier study^53^. Additionally, at high soil P supply (P50), the non-mycorrhizal *M*. *truncatula* plants were not as affected by the high soil Zn concentration, and this ‘alleviation’ of Zn toxicity at high P availability is possibly a result of dilution of Zn in the plant tissues due to the greater P uptake and, thus, biomass of the plants^63,71–74^.

### Medicago plants are more tolerant of Zn stress as deficiency or toxicity when colonised by AMF

In this study, there was evidence for the ‘dual roles’ of mycorrhizal fungi at low and at high soil Zn availabilities. When Zn was limiting to the plant, the mycorrhizal plants generally had higher shoot Zn contents; colonisation by *R*. *irregularis* also conferred a benefit to plants growing in toxic soil Zn conditions (Zn20), as indicated by the reduced shoot Zn concentration in the mycorrhizal plants at high soil P supply. Along with the increased biomass at Zn20 in the mycorrhizal plants at low soil P availability, these results support the hypothesis that mycorrhizal inoculation provides a ‘protective’ effect against high soil Zn availability. These results are in accordance with earlier studies exploring the benefits of mycorrhizal fungi in other plant species (red clover and white clover) in Zn-contaminated soil^29,75^. Additionally, other studies have indicated that under soil heavy metal contamination with high levels of plant-available soil P, mycorrhizal plants can increase their biomass, thus resulting in reduced uptake of heavy metals such as Zn, copper (Cu), and lead (Pb) into their tissues^28,70,72,76^.

### Expression of *ZIP* genes may be linked to mycorrhizal Zn tolerance mechanisms

The ZIP membrane transporter gene family can transport Zn^2+^ from the rhizosphere into plants via the roots^31^. There are four characterised ZIP transporters (MtZIP1, MtZIP2, MtZIP5 and MtZIP6) that are able to transport Zn^2+^ in *M*. *truncatula*, as confirmed by yeast complementation assays^37,38^.

Inoculation with AMF induced the expression of *MtZIP5* in Medicago roots when grown in Zn-deficient soil; this result has not to our knowledge been reported before. Given that the Medicago plants were growth-limited by Zn availability at Zn0, the induction of *MtZIP5* in mycorrhizal roots at all Zn0 treatments may have helped to overcome the Zn limitation somewhat, as supported by the greater biomass and Zn concentration. Therefore, as a Zn transporter, MtZIP5 may have a direct or indirect association with the MPU of Zn uptake, and this novel result is deserving of further study.

The expression of *MtZIP2* was down-regulated in the roots when inoculated with AMF, which is in line with an earlier study^39^. These results together suggest that MtZIP2 may not be directly involved in Zn uptake via the MPU. Similarly, the expression of the *MtZIP6* gene was also found to be down-regulated by AMF. By contrast, in a previous study, *MtZIP6* was highly up-regulated using the same plant and AMF species^53^; this difference may be due to differences in Zn and/or P availability in the two studies, or the different soil type used. The contrasting *MtZIP6* results with previous work, highlight that the role of the MtZIP6 protein may not be one directly involved in the MPU.

While the present study has shown that the expression of a number of *ZIP* genes are modified by AMF inoculation, further studies are required that use knock-out mutant plants in conjunction with radioisotope tracing to elicit the function of ZIP transporters, and their potential role in Zn uptake via the mycorrhizal pathway.

### Plant P nutrition was improved under P-deficiency and buffered under varying Zn-stress in mycorrhizal plants

The results of this study clearly confirm that mycorrhizal colonisation benefits Medicago plant tissue P concentration and contents at low soil P addition^13,54,71,77^. In addition, shoot P contents in mycorrhizal plants were maintained across the range of soil Zn availabilities (from deficient to toxic) at high soil P treatments (P20, P50). By contrast, the shoot P content in non-mycorrhizal plants was negatively influenced by soil Zn deficiency at high soil P concentration treatments, and is in agreement with a previous Zn-AMF interaction study in Medicago, up to 20 mg Zn kg^−1^ ^53^. However, once soil Zn reached a highly toxic concentration (40 mg Zn kg^−1^) in Watts-Williams, *et al*.^53^, mycorrhizal colonisation no longer had the capability to maintain plant shoot P contents. This ‘buffering’ capability of mycorrhizal plants at varying soil Zn concentrations when P is not deficient could be considered a benefit of AMF beyond simply increased plant P concentration and/or contents at P-deficiency.

### PT gene expression patterns are highly influenced by mycorrhizal colonisation

This study investigated the different roles of the phosphate (Pi) transport-related genes in *M*. *truncatula* plants and the effect of soil P availability and mycorrhizal inoculation. When plants are colonised by AMF, the expression of a number of membrane *PT* genes is modified. Specifically, two P transporters (MtPT4 and MtPT8) have been localised to the peri-arbuscular membrane (PAM), the site of plant-fungus nutrient exchange, and are directly implicated in the transport of P from fungus to the host plant^44,48^. In this study, *MtPT4* was exclusively expressed in mycorrhizal plants and was down-regulated with increasing soil P availability, with no influence of soil Zn availability. The lack of effect of Zn availability on *MtPT4* expression is in contrast with a previous study that reported that an increased expression of *MtPT4* corresponded with an increase in soil Zn concentration^53^. The present study suggests that *MtPT4* expression interacts with soil Zn availability under certain conditions only. Furthermore, in this study, the expression of *MtPT4* was highly correlated with the expression of *R*. *irregularis* *α-tubulin* (a mycorrhizal fungal biomass marker gene), as demonstrated in earlier studies^53,78^. Therefore, the induced expression of *MtPT4* at low soil P concentration may be due to the increased requirement for the MPU, and thus transport of P across the PAM, which in turn contributed to the higher plant P concentrations and contents in the mycorrhizal plants. Conversely, *MtPT8*, a second AMF-induced Pi transporter gene^47,48^, was highly expressed in the P20 treatment, where *MtPT4* had reduced expression. This interplay between the expression of *MtPT4* and *MtPT8* has been discussed in previous studies^48,49^ in which *MtPT4* was mutated, and this led to the compensation by *MtPT8* expression, presumably to balance the P homeostasis in plants colonised by AMF. This compensation by *MtPT8* in previous studies may also explain why *MtPT4* and *MtPT8* expression was dominant at different soil P availabilities in this study.

The expression of *PT* gene *MtPT1* is a representative of the direct pathway of P uptake (DPU), and here was down-regulated in the mycorrhizal plants across different soil P and Zn availabilities, which is consistent with previous studies^26,42^. The expression of *MtPT1* in non-mycorrhizal plants was particularly up-regulated when soil P was the most limiting to plants. This highlights again the important role that MtPT1 and other PTs may have in P uptake via the DPU in non-mycorrhizal plants, as they are relying on a single pathway of P uptake from the soil. Furthermore, the P starvation-induced gene *MT4*^50^ was also down-regulated in the mycorrhizal plants at low soil P availability; at any higher levels of soil P addition the expression of *MT4* was similar between mycorrhizal and non-mycorrhizal plants, presumably because the plant was not P-starved^26,51,79^. In summary, whereas *MtPT4* and *MtPT8* expression interacted with soil P availability in the mycorrhizal plants, *MtPT1* and *MtMT4* were likely important for P uptake and regulation in the non-mycorrhizal plants at low soil P availability.

### Soil P and Zn availabilities strongly influence responses to mycorrhiza and gene expression

This study demonstrated that, aside from inoculation with mycorrhizal fungi, soil Zn and P availability also have a powerful impact on plant physiology and gene expression. For example, in addition to the down-regulation effects of AMF on the expression of *MtZIP2*, this gene was also up-regulated by increased soil Zn addition. This finding has also been documented in Burleigh, *et al*.^39^, in which the authors observed that *MtZIP2* was up-regulated within roots by high Zn fertilisation, and was highest in roots exposed to a toxic level of soil Zn. Thus, it is possible that *MtZIP2* is either expressed directly in response to high concentrations of soil Zn, or is stimulated by internal plant Zn concentrations. Given that both shoot Zn concentrations and the expression of *MtZIP2* were generally lower in the mycorrhizal plants, the results presented here suggest that the second hypothesis is more plausible.

By contrast, expression of *MtZIP5* in roots appeared to be soil Zn deficiency-induced, which has not been shown previously; this result suggests that a threshold in soil or plant Zn concentration exists between the Zn0 and Zn5 treatments, and thus that *ZIP5* expression was reduced to a baseline level when the plant was not considered Zn-limited. A similar trend was also identified in barley whereby the expression of six *HvZIP* genes was increased by at least three-fold in Zn-deficient roots compared to the expression in Zn-sufficient plant roots^34^.

In a previous study, researchers observed that loss-of-function of *LPCAT1* in *Arabidopsis thaliana* at soil Zn-deficient conditions lead to increased shoot P accumulation^52^. Therefore, this recent study hypothesised that the expression of the nearest orthologue of *LPCAT1* in Medicago may be highly interactive with soil P and Zn concentration. However, the expression of this gene was not affected by the interaction between soil P and Zn availability, although expression was higher in mycorrhizal plants than in non-mycorrhizal plants across all soil Zn addition treatments. Therefore, the role of this gene in Medicago, and other species with the ability to form arbuscular mycorrhizal associations remains unclear, but we have in this study uncovered a potential interaction between the gene and mycorrhizal inoculation.

### Conclusions and future research

This experiment presents, to our knowledge, the first attempt to link physiological and molecular markers of mycorrhizal associations pertaining to both Zn and P nutrition, in Medicago. The expression of *MtZIP5* was induced both by mycorrhizal colonisation and low soil Zn availability. In contrast, *MtZIP2* expression was up-regulated in non-mycorrhizal roots, and increased with soil Zn availability. In examining shoot biomass and Zn concentration, there was evidence of a ‘protective’ role of mycorrhizal fungi at high levels of soil Zn. Regarding PTs, *MtPT4* and *MtPT8* were up-regulated, and *MtPT1* and *MtMT4* down-regulated in mycorrhizal plants; the expression of all PTs was interactive with available soil P. The expression of both MPU- and DPU-related PTs likely conferred greater P uptake in the mycorrhizal plants when soil P was limiting. Further studies are necessary to understand the potential of mycorrhizal fungi and the role of ZIP transporters to improve the Zn nutrient uptake via the MPU.

## Supplementary information

Supplementary information

## Data Availability

The datasets generated during and/or analysed during the current study are available from the corresponding author on reasonable request.
