# Supplementary material for: The effects of soil phosphorus and zinc availability on plant responses to mycorrhizal fungi: a physiological and molecular assessment
Source: Sci Rep. 2019 Oct 16;9:14880. doi: 10.1038/s41598-019-51369-5 (PMC6795859; doi:10.1038/s41598-019-51369-5)
Supplement: Supplementary file 1 — Supplementary information [file 41598_2019_51369_MOESM1_ESM.pdf]

Supplementary information

The effects of soil phosphorus and zinc availability on plant responses to mycorrhizal fungi: a physiological and molecular assessment

Authors: Thi Diem Nguyen, Timothy R. Cavagnaro, Stephanie J. Watts-Williams

Supplementary information

Table 1. The forward and reverse oligo sequences used to amplify housekeeping genes and genes-of-interest, gene ID (Phytozome) and brief description of genes.

Gene name	Forward primer	Reverse primer	Gene ID	Gene
MtEF1α	TGACAGGCGATCTGGTAAGG	TCAGCGAAGGTCTCAACCAC	Medtr6g021800	Housekeeping
MtASPP	GGATCGGTCTTGGACAGTGG	TGGACCGCTGATTTGACTGA	Medtr4g095270	Housekeeping
MtTRF	GGATAAGGTGGATGGTGATCG	TCTGCCTCTCGTCGTTTTTGT	Medtr1g087790	Housekeeping
MtZIP2	AATGGGCATTGCTTGTGGTG	TGTCGAAACGGCTCTTCCTC	Medtr8g105030	ZIP
MtZIP5	GCGTCGCAACATGGACATTC	CGGACGCTACTATCCACACC	Medtr2g097580	ZIP
MtZIP6	AGGACTTGGAATGGGAGCCT	CACCAAGACCCATGCCTTCA	Medtr1g016120	ZIP
MtPT1	AGCCCGTTACACCGCTCTTG	CATGTTCCAAACAAAGCCAAACCG	Medtr1g043220	PT
MtLPCAT1	TGGCTTAACAGCACGTTGGA	CTGAAACCCAAGCCGGAGAT	Medtr4g102750.1	?
MtMT4	CCTTTATGGAACCACATGGGA	AAGAAGGTTCATTCCCAGCAA	Medtr1g062300.1	PSI
Ri a-tubulin	TGTCCAACCGGTTTTAAAGT	AAAGCACGTTTGGCGTACAT	TC105406	Fungal biomass
MtPT8	GCTGCAGCTGGAAAATCAGG	CAAGTTCACAACCGCAAGGG	Medtr5g068140.1	PT
MtPT4	GACACGAGGCGCTTTCATAGCAGC	GTCATCGCAGCTGGAACAGCACCG	Medtr1g028600	PT

(Housekeeping genes from Bravo, et al. [81], ZIP genes from Watts-Williams, et al. [53])
